# Development and Internal Validation of a Bailout Risk Score in PCI with Drug-Coated Balloons

**DOI:** 10.3390/jcm15020813

**Published:** 2026-01-19

**Authors:** Luigi Alberto Iossa, Marco Ferrone, Luigi Salemme, Elena Laganà, Armando Pucciarelli, Michele Franzese, Giuseppe Ciliberti, Sebastiano Verdoliva, Giulia Sgherzi, Grigore Popusoi, Angelo Cioppa, Tullio Tesorio, Giuseppe Di Gioia

**Affiliations:** 1 Division of Interventional Cardiology, Montevergine Clinic, 83013 Mercogliano, Italy; luigi.alberto.iossa@gmail.com (L.A.I.); marco.ferrone1@gmail.com (M.F.); ginosalemme@hotmail.it (L.S.); armandopucciarelli@gmail.com (A.P.); mik.franzese@gmail.com (M.F.); giuseppeciliberti6@gmail.com (G.C.); dr.verdoliva@gmail.com (S.V.); gsgherzi@gmail.com (G.S.); gpopusoi@yahoo.com (G.P.); cioppa68@gmail.com (A.C.); tulliotesorio@gmail.com (T.T.); 2 Division of Cardiology, Department of Medicine, University of Verona, Piazzale A. Stefani 1, 37126 Verona, Italy; elenalagana98@gmail.com; 3 Department of Advanced Biomedical Sciences, Federico II University of Naples, 80131 Naples, Italy

**Keywords:** drug-coated balloon, percutaneous coronary intervention, bailout stenting, risk stratification, predictive risk score, coronary artery disease

## Abstract

**Background/Objectives**: Bail-out stenting remains a procedural challenge for percutaneous coronary intervention (PCI) performed with drug-coated balloons (DCBs). No dedicated bedside tool is currently available to predict this event. We aimed to develop and internally validate a bedside Bail-Out Risk Score. **Methods**: We analyzed patients treated with DCBs between 2021 and 2025. Predictors of bailout stenting were identified through univariate analysis, and variables with *p* < 0.10 were entered into a multivariable logistic regression model. Regression coefficients were then transformed into integer points using the Sullivan method. Model performance was evaluated by AUC-ROC, calibration, and bootstrap internal validation (B = 1000). **Results**: A total of 352 patients (399 de novo lesions) were treated with DCB-only PCI. Bail-out stenting occurred in 14.5% of lesions (58/399). Independent predictors of bail-out stenting were prior CABG (OR 4.29, *p* = 0.002), proximal lesion location (OR 2.99, *p* = 0.003), and diffuse disease (OR 2.18, *p* = 0.018). Prior PCI (OR 0.44, *p* = 0.009) and lipid-lowering therapy (OR 0.42, *p* = 0.029) were protective, while LAD involvement showed a non-significant trend (OR 1.57, *p* = 0.137). The model demonstrated moderate discrimination (AUC = 0.734; optimism-corrected AUC = 0.704) and excellent calibration (intercept = 0.000, slope = 1.000). The final score (range −4 to +8) stratified lesions into low (≤−1), intermediate (0–3), and high (≥3) risk groups, with progressively higher predicted probabilities (≤9%, 13–37%, and ≥49%). **Conclusions**: The Bail-Out Risk Score provides a practical and reliable bedside tool to estimate procedural risk during stentless PCI.

## 1. Introduction

Over the past decade, drug-coated balloons (DCBs) have emerged as a valid alternative to conventional stent implantation in percutaneous coronary intervention (PCI). By delivering an antiproliferative drug directly to the vessel wall without leaving a permanent metallic scaffold, DCBs enable a “leave-nothing-behind” strategy that preserves vessel physiology and potentially reduces long-term complications such as stent thrombosis, neoatherosclerosis, and late restenosis [1,2,3].

DCB angioplasty currently represents one of the treatments of choice for in-stent restenosis (ISR) according to current European guidelines [4,5]. More recently, this technology has gained increasing adoption in de novo coronary lesions, particularly in small vessels, bifurcations, and in patients with a high bleeding risk, in which avoiding permanent implants and prolonged dual antiplatelet therapy provides a clinical advantage [6,7].

Despite these benefits, the DCB-only approach remains technically demanding. The success of the procedure depends on careful lesion preparation, optimal vessel sizing, and adequate drug transfer during balloon inflation. Achieving an optimal angiographic result—characterized by absence of significant recoil or dissection—is crucial for procedural safety and long-term patency [8]. When this condition is not met, unplanned stent implantation (“bailout stenting”) may become necessary to restore flow or seal a dissection flap [9].

Bailout stenting effectively negates the core advantage of the stentless approach. Reported rates of bailout vary widely, ranging from 5% to 20%, largely depending on lesion characteristics, operator experience, and procedural selection [10,11,12]. In recent studies—conducted exclusively in the setting of DCB angioplasty for small-vessel disease—independent predictors of bailout stenting included a high DCB-to-reference vessel diameter (RVD) ratio, marked vessel tortuosity, distal vessel involvement, and ACC/AHA Grade C lesion [13]. Moreover, lesions involving the left anterior descending artery (LAD) are often approached with greater caution due to the extent of myocardium at risk [9].

A growing body of evidence has attempted to characterize predictors of bailout stenting. However, these have been limited to observational associations and lack integration into a quantitative, clinically usable model. Operators currently rely on qualitative visual assessment or intuition rather than structured risk estimation [9,13]. Consequently, the decision to attempt a stentless strategy remains largely subjective, which may hinder procedural planning and patient counseling.

A dedicated risk score capable of predicting the likelihood of bailout stenting could therefore fill an important gap in interventional cardiology. Such a tool could achieve the following:

1. Help identify patients or lesions at high risk of bailout, allowing the operator to proceed directly with stent implantation when appropriate—thereby saving time, reducing procedural costs, and avoiding unnecessary DCB usage;
2. Support better procedural planning, enabling operators to anticipate the potential need for bailout and predefine the type and size of stent to be used if required;
3. Improve communication and informed consent by providing an objective and quantifiable estimate of procedural risk;
4. Facilitate training, quality assurance, and research standardization in stentless revascularization.

The present study was therefore designed to develop and internally validate a simple, bedside Bailout Risk Score for DCB-only PCI in de novo coronary lesions. Through systematic evaluation of clinical and angiographic predictors, we aimed to identify the key determinants of bailout stenting and transform them into a practical, point-based tool for use in everyday practice.

## 2. Materials and Methods

### 2.1. Study Population

Consecutive patients with de novo coronary lesions who underwent drug-coated balloon (DCB)–only percutaneous coronary intervention (PCI) with the Selution SLR™ Sirolimus-coated balloon at Montevergine Clinic (Mercogliano, Italy) between March 2021 and July 2025 were included in the study. All procedures were performed according to institutional protocols and contemporary European recommendations for DCB angioplasty. Procedures involving in-stent restenosis (ISR) or hybrid revascularization (combined use of DCB and stent implantation on the same lesion) were excluded. Interventions performed on bypass grafts were also excluded, although patients with a history of previous CABG were eligible for inclusion if the treated lesion was in a native coronary vessel. The study was conducted in accordance with the ethical principles of the Declaration of Helsinki and was approved by the local institutional ethics board. Written informed consent was obtained from all patients.

### 2.2. Outcome Definition

The dependent outcome for model derivation was the occurrence of bailout stenting, defined as any unplanned stent implantation on the target lesion in the presence of significant residual stenosis (>30%), flow-limiting dissection (National Heart, Lung, and Blood Institute [NHBLI] ≥ Type C), or vessel closure following DCB inflation.

This variable was used to identify independent predictors and construct a point-based Bailout Risk Score applicable to DCB-only PCI.

### 2.3. Data Collection and Variables

Comprehensive clinical profiles, lesion characteristics, and procedural parameters were prospectively entered into a dedicated institutional database specifically developed for the systematic evaluation of DCB PCI cases. Clinical variables included age, sex, cardiovascular risk factors, prior myocardial infarction (MI), previous PCI or coronary artery bypass grafting (CABG), and renal function. Lesion characteristics encompassed vessel location, segment (proximal vs. mid/distal), morphology (focal vs. diffuse) and calcification grade. Procedural features included DCB size, inflation pressure, and use of intravascular imaging. The threshold between focal and diffuse disease was set at 20 mm, in accordance with previous studies that defined focal disease as a significant pressure drop occurring within ≤20 mm [14,15].

### 2.4. Statistical Analysis

Continuous variables are presented as mean and standard deviation if normally distributed or with median value and interquartile range otherwise. Continuous variables were compared with unpaired *t*-test, one-way ANOVA or the Mann–Whitney test as appropriate. Categorical data are reported as numbers (percentages) and compared with the χ^2^ test or Fisher’s exact test as appropriate. All analyses were performed using Jamovi^®^ software (version 2.5.7, @jamoviStats, Sydney, Australia) and its integrated R editor environment for advanced modeling and graphical analyses.

Univariate logistic regression was first performed to identify factors associated with the occurrence of bailout stenting. Variables showing a *p*-value < 0.10 were subsequently entered into the multivariate analysis to ensure inclusion of all potential predictors. A backward elimination strategy was then applied to retain only those variables demonstrating independent or borderline significance.

Model performance was evaluated using the area under the receiver operating characteristic curve (AUC) to assess discrimination. The AUC was computed using the rank-sum method. Model calibration was evaluated through the calibration slope and intercept, and further examined graphically by plotting predicted versus observed event rates across deciles of predicted risk.

To account for optimism and potential overfitting, bootstrap internal validation was performed with 1000 resamples. Optimism-corrected AUC values and corresponding 95% confidence intervals were then derived to provide a robust estimate of model discrimination.

Regression coefficients (β) from the final multivariable logistic regression model—corresponding to the log-transformed odds ratios (log OR) of the independent predictors—were used to construct the Bailout Risk Score according to the Sullivan method [16].

Briefly, the smallest absolute β coefficient among the significant predictors was selected as the scaling factor, ensuring that each variable contributed proportionally to the total score.

Each β coefficient was divided by this scaling factor and rounded to the nearest integer to obtain the point value assigned to that predictor.

Positive weights were attributed to variables associated with an increased likelihood of bailout stenting (β > 0; OR > 1), whereas negative weights were assigned to protective variables (β < 0; OR < 1).

The total Bailout Risk Score was then calculated as the sum of all weighted predictors, with higher scores indicating a greater estimated probability of bailout stenting during DCB-only PCI.

The predicted probability of bailout stenting was estimated according to the logistic regression equation:

$$P\left(bail-out\right)=\frac{1}{1+e^{\left(\alpha \;+\;\beta \;\times \;Total\;Score\right)}}$$

where α represents the model intercept, while β corresponds to the scaling factor derived from the original multivariable model, reflecting the incremental change in the log-odds of the outcome per one-point increase in the total risk score.

Predicted probabilities derived from this equation were used to stratify patients according to increasing estimated risk.

Model performance was further assessed through the area under the ROC curve (AUC) for discrimination and calibration plots comparing predicted and observed event rates across risk deciles.

To define the optimal threshold between low and higher-risk categories, the Youden Index was applied to the receiver operating characteristic (ROC) curve, identifying the point that maximized the sum of sensitivity and specificity.

## 3. Results

### 3.1. Baseline and Angiographic Characteristics

Overall, 352 patients with 399 de novo coronary lesions were included in the study. The main baseline characteristics are reported in Table 1. The mean age was 68.5 ± 9.1 years, and 78.4% were male. Most procedures were performed for chronic coronary syndrome, while only five lesions were treated in the setting of acute coronary syndrome. LAD was the most frequently treated vessel (49.7%), followed by the RCA (29.3%) and LCx (21.0%).

Bailout stenting occurred in 58 lesions (14.5%), most commonly due to flow-limiting dissection (70.7%). Residual stenosis >30% and vessel closure accounted for 27.6% and 1.7% of cases, respectively.

Compared with lesions successfully treated with DCB-only PCI, those requiring bailout stenting more frequently involved patients with a history of prior CABG (15.5% vs. 5.2%, *p* = 0.004) and less often prior PCI (31.0% vs. 52.5%, *p* = 0.003). Similarly, lipid-lowering therapy was less prevalent among patients who required bailout stenting (79.3% vs. 88.8%, *p* = 0.042). No significant differences were observed in age or sex between groups. From an angiographic standpoint, lesions that required bailout were longer on average than those successfully treated with DCB only (31.9 ± 15.0 mm vs. 27.7 ± 14.4 mm, *p* = 0.043). Angiographic characteristics are reported in Table 2.

### 3.2. Univariate Analysis

At univariate analysis, several clinical and angiographic factors showed a significant association with the occurrence of bailout stenting (Supplementary Table S1).

At univariable analysis, a history of prior CABG was associated with a significantly higher likelihood of unplanned stent implantation (OR 3.30, 95% CI 1.40–7.75, *p* = 0.006). Lesions located in proximal segments and those with a diffuse morphology were also associated with an increased risk of bailout stenting (OR 2.48, 95% CI 1.27–4.86, *p* = 0.008 and OR 1.96, 95% CI 1.07–3.59, *p* = 0.029, respectively).

Conversely, lipid-lowering therapy, a history of prior PCI, and prior myocardial infarction were each associated with a significantly lower probability of bailout (OR 0.48, 95% CI 0.23–0.99, *p* = 0.046; OR 0.41, 95% CI 0.23–0.74, *p* = 0.003; and OR 0.09, 95% CI 0.01–0.67, *p* = 0.019, respectively).

Treatment of lesions in the left anterior descending artery showed a non-significant trend toward a higher risk of bailout (OR 1.64, 95% CI 0.94–2.88, *p* = 0.082), as did the use of intravascular imaging (OR 3.67, 95% CI 0.85–15.77, *p* = 0.081). No significant associations were observed for the remaining clinical or procedural variables.

### 3.3. Multivariate Analysis

A multivariate logistic regression model was constructed, including only the candidate variables that emerged from univariate testing with a *p*-value < 0.10.

Given the limited number of events (58 bailout stentings) and following the conventional rule of one predictor per ten events, prior myocardial infarction was not considered to avoid model overfitting. Since prior PCI implicitly captures previous ischemic disease, it was retained instead.

In the initial multivariable model, intravascular imaging was not independently associated with bailout stenting (OR 2.57, 95% CI 0.54–12.30, *p* = 0.238) and was therefore excluded from the final model.

In the final multivariable analysis, five independent predictors were retained. Prior CABG was associated with a markedly increased risk of bailout stenting (OR 4.29, 95% CI 1.70–10.80, *p* = 0.002). Proximal lesion location was also independently associated with higher odds of bailout (OR 3.00, 95% CI 1.46–6.14, *p* = 0.003), as was diffuse lesion morphology compared with focal disease (OR 2.18, 95% CI 1.15–4.14, *p* = 0.018).

**Figure 1:**
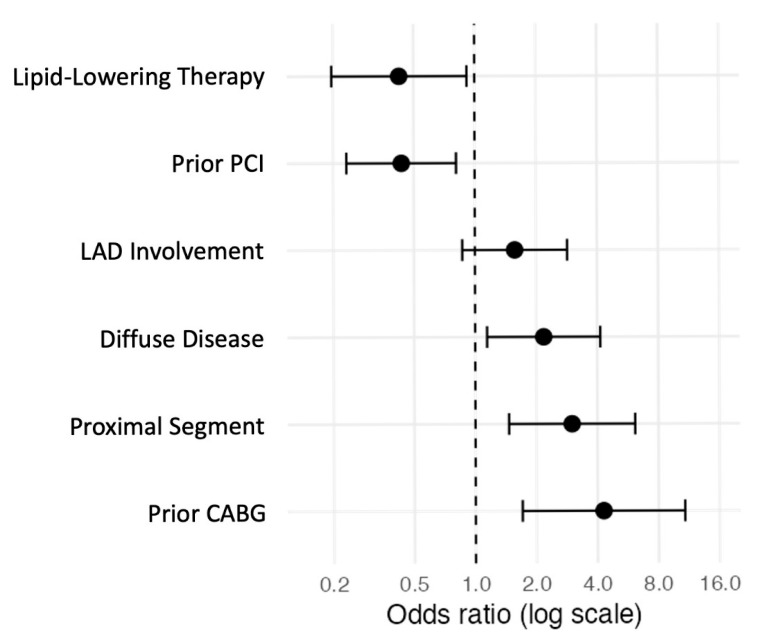
Forest plot showing the independent predictors of bailout stenting during DCB-only PCI identified through multivariable logistic regression analysis. Dots represent odds ratios, with horizontal lines indicating 95% confidence intervals. The vertical dotted line represents the line of no effect (odds ratio = 1).

Conversely, prior PCI and lipid-lowering therapy were both independently associated with a lower probability of bailout stenting (OR 0.44, 95% CI 0.23–0.81, *p* = 0.009 and OR 0.42, 95% CI 0.20–0.92, *p* = 0.029, respectively). LAD involvement did not reach statistical significance in the adjusted analysis (OR 1.57, 95% CI 0.87–2.85, *p* = 0.137). Although LAD involvement did not reach statistical significance in the final multivariable model, it was retained in the risk score due to its strong clinical relevance and consistent directional effect across analyses (Figure 1).

The model intercept (α) was −1.763, corresponding to the constant term used in the logistic equation for predicted probability estimation.

No evidence of multicollinearity was observed among the predictors (all VIF values < 1.1 and tolerance > 0.90), confirming that the included variables were statistically independent and suitable for multivariable modeling.

### 3.4. Calibration and Internal Validation

Calibration was excellent, with an intercept of 0.000 and a slope of 1.000, indicating no systematic over- or underestimation of predicted probabilities. Calibration across deciles demonstrated a close agreement between observed and predicted risks. In the lowest risk decile, the observed event rate was 2.5% compared with 3.1% predicted, while in the highest decile, observed and predicted risks were 43.6% and 41.1%, respectively. These findings indicate that the model maintained accurate calibration across the full spectrum of predicted probabilities, particularly in the high-risk range (Supplementary Figure S1).

**Figure 2:**
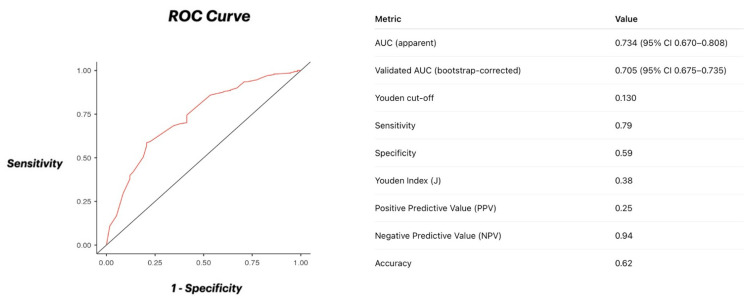
ROC Curve. Receiver-operating characteristic (ROC) curve showing the discriminative ability of the Bailout Risk Score model for predicting bailout stenting.

Internal validation through 1000 bootstrap resamples confirmed the model’s stability and discriminative performance, yielding an apparent AUC of 0.734 and an optimism-corrected AUC of 0.704 (95% CI 0.675–0.735) after 1000 bootstrap replications. The optimal cut-off (0.130) identified at the Youden point corresponded to 79% sensitivity, 59% specificity, ~25% positive predictive value, ~94% negative predictive value, and ~62% accuracy (Figure 2).

### 3.5. Development of the Bailout Risk Score

The Bailout Risk Score was developed using the Sullivan method, which converts regression coefficients from a logistic model into a simplified, point-based system for clinical application.

First, the natural logarithms of the odds ratios (log ORs) from the final multivariable model were computed to obtain the regression coefficients (βᵢ). These coefficients quantify the log-odds change in the probability of bailout stenting associated with each independent predictor.

The smallest absolute β value among the significant predictors was selected as the scaling factor (β = 0.452). Each β coefficient was then divided by this scaling factor and rounded to the nearest integer to determine the relative weight of each variable.

Accordingly, the following point assignments were derived: Prior CABG: +3 points; LAD involvement: +1 point; Proximal lesion segment: +2 points; Diffuse disease morphology: +2 points; Prior PCI (on other vessels): −2 points (protective); Lipid-Lowering Therapy: −2 points (protective).

Positive points corresponded to risk-enhancing factors, whereas negative points were attributed to protective variables. The resulting composite score ranged from −4 to +8, with higher scores indicating progressively greater procedural risk.

Finally, the predicted probability of bailout stenting for each total score was calculated by substituting the score values into the logistic regression equation:

$$P\left(bail-out\right)=\frac{1}{1+e^{\left(\alpha \;+\;\beta \;\times \;Total\;Score\right)}}$$

where the intercept (α) was approximately −1.764 and the slope (β) was 0.452, representing the incremental change in log-odds per one-point increase in the total score.

**Figure 3:**
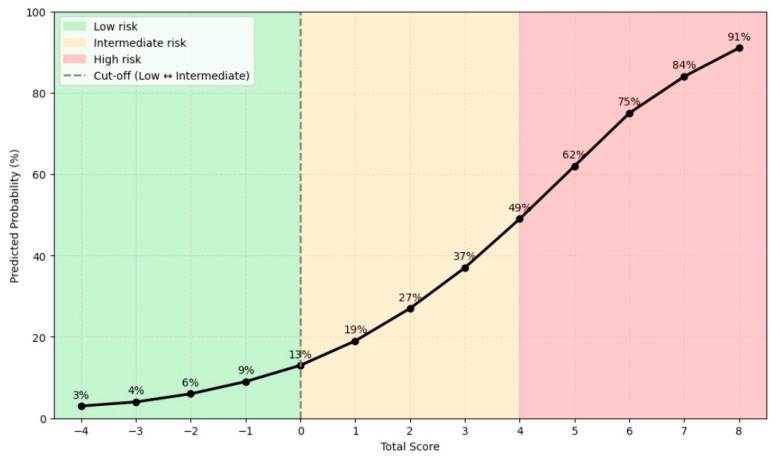
Nomogram-derived Predicted Probability of Bailout Stenting.

Insertion of the various score values into this equation produced a progressive increase in predicted risk, ranging from 3% for −4 points to 91% for +8 points, demonstrating a consistent and monotonic risk gradient (Figure 3).

Graphical representation of the Bailout Risk Score showing the progressive increase in predicted probability of bailout stenting across the total score range (−4 to +8). The dashed line marks the Youden index, which effectively separates lesions with a low likelihood of bailout stenting from those with a higher procedural risk.

### 3.6. Risk Stratification

Based on Youden’s index, the optimal cut-off for discrimination between low- and higher-risk categories was identified at a predicted probability of 0.130, corresponding to a total score of 0. The Youden index provided a dichotomous distinction between lower- and higher-risk lesions.

For greater clinical interpretability, the higher-risk group was further subdivided into intermediate and high-risk categories, according to the progressive increase in predicted probability. This additional stratification was guided by clinical judgment and intuitive reasoning, rather than by a formal statistical criterion, to better capture the continuous gradient of procedural risk observed in real-world DCB-only PCI.

**Figure 4:**
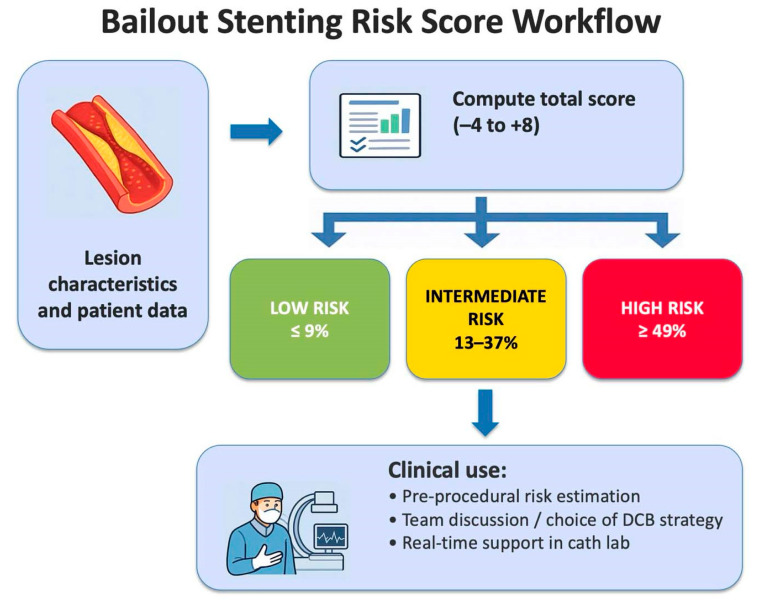
Bailout Stenting Risk Score Workflow.

The model demonstrated a clear stepwise risk gradient, supporting its potential value as a bedside decision-making tool during DCB-only interventions (Figure 4).

## 4. Discussion

This study presents the first dedicated and internally validated risk score for predicting bailout stenting during DCB-only PCI in de novo coronary lesions. Using six readily available clinical and angiographic parameters, we derived a simple, point-based tool that showed moderate discrimination and excellent calibration.

The Bailout Risk Score integrates both lesion-related and clinical determinants of procedural failure. Among all predictors, prior CABG emerged as the strongest independent determinant of bailout stenting. Although graft interventions were excluded from the analysis, patients with a history of bypass surgery likely represent a subgroup with diffuse and functionally advanced coronary atherosclerosis. Physiologic studies have shown that native vessels in post-CABG patients often exhibit lower fractional flow reserve (FFR) or vessel FFR (vFFR) values and a more heterogeneous pressure profile, even in angiographically moderate segments, reflecting extensive endothelial dysfunction and microvascular disease progression [17]. These alterations may translate into impaired vessel compliance and higher susceptibility to recoil or flow-limiting dissections despite adequate lesion preparation. Therefore, the association between prior CABG and the need for bailout stenting likely reflects the intrinsic vulnerability of the native coronary circulation rather than procedural factors related to graft anatomy. In line with prior physiologic studies, this finding supports the notion that post-CABG native arteries behave as diffusely diseased, fragile conduits, in which achieving durable luminal expansion with a purely stentless approach remains technically and biologically challenging.

Proximal lesions were also associated with a higher likelihood of bailout stenting. In these large-caliber and hemodynamically relevant segments, flow-limiting dissections were less tolerated, as they were more likely to cause significant ischemia or vessel compromise, prompting immediate stent implantation to secure patency. Conversely, in distal vessel segments, operators often adopted a more conservative strategy, allowing brief observation of transient flow limitation, given that distal dissections often stabilized without the need for bailout stenting.

Diffuse disease was also associated with a higher risk of bailout stenting. This finding may reflect several procedural and anatomical challenges typically observed in this setting, including the need for longer balloon coverage, heterogeneous plaque composition, and reduced vessel compliance. In addition, the cumulative effect of multiple microdissections along the treated segment may contribute to a greater likelihood of flow-limiting injury, ultimately requiring stent implantation to restore lumen integrity.

The inclusion of LAD involvement as a borderline factor is clinically plausible: although not statistically significant, the LAD remains a critical vessel where operators are more reluctant to accept dissections, especially when large territories are at risk.

Conversely, prior PCI and lipid-lowering therapy were associated with a lower likelihood of bailout stenting. Prior PCI may identify patients with more stable coronary anatomy and optimized secondary prevention, whereas ongoing lipid-lowering therapy, such as chronic statin use, has been associated with improved plaque stability, reduced periprocedural inflammation, and enhanced vascular healing, ultimately favoring procedural success with a stentless strategy [18,19,20].

Previous reports on DCB-only PCI have mainly focused on technical outcomes, late lumen loss, or long-term MACE. Bailout stenting has been treated as a secondary procedural event, without systematic modeling of predictors.

To date, two studies have specifically evaluated the predictors of bailout stenting during DCB angioplasty. The first, conducted by Ghetti et al. [13], analyzed de novo lesions but was limited to small-vessel disease, identifying several clinical and angiographic correlates of unplanned stent implantation without developing or validating a dedicated risk model. The second, a subanalysis of the Eastbourne Study by Gurgoglione et al. [12], investigated bailout stenting in sirolimus-coated balloon PCI but did not differentiate between in-stent restenosis and de novo lesions, thereby limiting its applicability to the present context [12,13]. In contrast, our analysis derived and internally validated a pragmatic, point-based Bailout Risk Score capable of estimating the likelihood of unplanned stent implantation before DCB-only PCI, thus providing a simple bedside tool to support patient selection and procedural planning. The moderate discriminative ability (AUC ≈ 0.70) is comparable to early versions of widely used interventional scores (e.g., SYNTAX-II, ACUITY), which later improved with multicenter validation. The optimal cut-off (0.130) identified at the Youden point corresponded to 79% sensitivity, 59% specificity, ~25% positive predictive value, ~94% negative predictive value, and ~62% accuracy, indicating that the model is particularly reliable in ruling out patients at low risk of bailout stenting. This performance suggests that, while the score may overestimate risk in borderline cases, it effectively discriminates those who can safely undergo a stentless strategy, minimizing unnecessary device implantation.

Model performance was consistent with expectations for a single-center derivation cohort, with an optimism-corrected AUC of 0.704, reflecting moderate discriminatory ability and good internal stability after 1000 bootstrap replications.

Calibration analysis demonstrated excellent agreement between predicted and observed probabilities across risk strata (intercept 0.000, slope 1.000), supporting the robustness of the model.

Moreover, the use of the Sullivan method allowed simple point conversion of logistic coefficients, translating a complex statistical model into an intuitive, bedside tool that can be applied in daily clinical decision-making.

**Figure 5:**
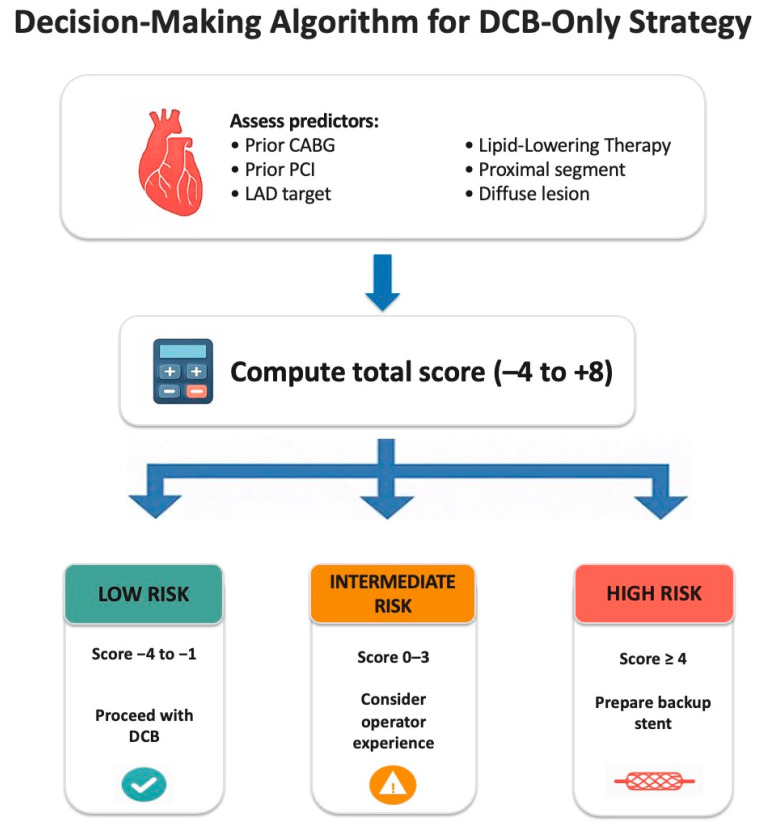
Cath-lab flow chart for procedural strategy selection during DCB-only PCI.

The score can be applied a priori during procedural planning to identify lesions with low, intermediate, or high risk of bailout. For low-risk lesions (≤−1 point), operators may confidently pursue a stentless approach, whereas high-risk cases (≥4 points) may warrant preparation for potential stent implantation or even reconsideration of a DCB-only strategy. Beyond improving procedural safety and patient counseling, this structured assessment may also offer practical and economic advantages. By anticipating the likelihood of bailout, operators can save procedural time by opting directly for stenting in high-risk lesions and avoid unnecessary DCB inflation in lesions predicted to respond poorly, thereby reducing overall procedural costs and improving resource efficiency. Ultimately, systematic risk stratification could enhance workflow, optimize resource utilization, and standardize lesion selection in future DCB registries and clinical trials. A cath-lab flow chart was included to simplify decision-making and assist operators in selecting the most appropriate revascularization strategy during the procedure (Figure 5).

Despite its strengths, this study has several limitations that should be acknowledged. It was a single-center, retrospective analysis, which may restrict external generalizability. Although internal validation through 1000 bootstrap resamples was robust, external validation in independent, multicenter cohorts will be essential to confirm model reproducibility and clinical applicability. The sample size, while adequate for model derivation, may have been underpowered to detect infrequent predictors. Procedural parameters such as balloon sizing and inflation duration were not standardized, reflecting real-world practice but potentially introducing operator-dependent variability. Moreover, quantitative coronary angiography (QCA) was not systematically performed, and reference vessel diameter was assessed visually, possibly introducing measurement bias. Vessel tortuosity was not coded in the dataset and therefore could not be analyzed as a potential anatomical determinant of bailout. Additionally, acute coronary syndromes (ACS) were rarely represented in the study cohort, which limits the generalizability of the model to acute clinical settings, where lesion instability and procedural dynamics may differ substantially. Looking ahead, expanding the score’s validation to multicenter datasets will be crucial. Integration into electronic reporting systems could enable real-time risk computation during PCI, while coupling the model with procedural imaging data or artificial intelligence–based lesion analysis may further enhance its precision and clinical utility.

## 5. Conclusions

In this single-center study, we developed and internally validated a simple Bailout Risk Score to predict unplanned stent implantation during DCB-only PCI. The model, based on six independent predictors, demonstrated moderate discrimination and excellent calibration. By integrating readily available clinical and angiographic information, the score enables stratification of DCB-treated lesions into low, intermediate, and high risk of bailout. This practical tool supports procedural decision-making and could facilitate more confident adoption of stentless strategies in contemporary interventional cardiology.

## Figures and Tables

**Table 1:** Baseline Clinical Characteristics According to the Occurrence of Bailout Stenting.

Variable	Overall (n = 399)	Bailout No (n = 341, 85%)	Bailout Yes (n = 58, 15%)	*p*-Value
Age (years)	68.5 ± 9.1	68.4 ± 9.26	68.7 ± 9.29	0.844
Male sex	313 (78.4%)	268 (78.5%)	45 (77.5%)	0.863
Hypertension	342 (85.7%)	293 (85.9%)	49 (84.4%)	0.772
Lipid-Lowering Therapy	349 (87.4%)	303 (88.8%)	46 (79.3%)	0.042
Diabetes	164 (41.1%)	136 (39.8%)	28 (48.2%)	0.230
Current smoker	88 (22.0%)	78 (22.8%)	10 (17.2%)	0.339
Prior CABG	27 (6.7%)	18 (5.2%)	9 (15.5%)	0.004
Prior PCI	197 (49.3%)	179 (52.5%)	18 (31.0%)	0.003
Previous MI	56 (14.0%)	55 (16.1%)	1 (1.7%)	0.004
CKD	90 (22.5%)	81 (23.7%)	9 (15.5%)	0.165
LVEF (%)	53.4 ± 6.3	53.2 ± 6.4	54.3 ± 5.3	0.211
ACS	5 (1.2%)	4 (1.1%)	1 (1.7%)	0.681
STEMI	1 (0.2%)	1 (0.3%)	0 (0%)	0.650
Stable CAD	393 (98.4%)	337 (98.8%)	56 (96.5%)	0.670

CABG, coronary artery bypass grafting, PCI, percutaneous coronary intervention, MI, myocardial infarction, CKD, chronic kidney disease, LVEF, left ventricular ejection fraction, ACS, acute coronary syndrome, STEMI, ST-elevation myocardial infarction, CAD, coronary artery disease.

**Table 2:** Angiographic Characteristics According to the Occurrence of Bailout Stenting.

	Overall (n = 399)	Success (n = 341, 85%)	Bailout Stenting (n = 58, 15%)	*p*-Value
Target vessel				0.209
LAD	178 (44.6)	146 (42.8)	32 (55.1)	
LCX	51 (12.7)	47 (13.7)	4 (6.8)	
RCA	117 (29.3)	101 (29.6)	16 (27.5)	
Diagonal	20 (5.0)	17 (4.9)	3 (5.1)	
Obtuse Marginal	28 (7.0)	25 (7.3)	3 (5.1)	
Ramus Intermedius	5 (1.2)	5 (1.4)	0 (0.0)	
Vessel Segment				0.052
Proximal	57 (14.2)	42 (12.3)	15 (25.8)	
Mid	185 (46.3)	160 (46.9)	25 (43.1)	
Distal	104 (26.0)	92 (26.9)	12 (20.6)	
Side Branch	53 (13.2)	47 (13.7)	6 (10.3)	
Calcification				0.436
Mild	250 (62.6)	218 (63.9)	32 (55.1)	
Moderate	110 (27.5)	91 (26.6)	19 (32.7)	
Severe	39 (9.7)	32 (9.3)	7 (12.0)	
Lesion Length, mm	28.3 ± 14.6	27.7 ± 14.4	31.9 ± 15.0	0.043
Maximal Lesion Size, mm	2.71 ± 0.44	2.70 ± 0.43	2.79 ± 0.47	0.147

LAD, left anterior descending, LCX, left circumflex, RCA, right coronary artery.

## Data Availability

The data that support the findings of this study are not publicly available due to their potential use in future related publications. Reasonable requests for access may be considered by the corresponding author.

## Supplementary Materials

The following supporting information can be downloaded at: https://www.mdpi.com/article/10.3390/jcm15020813/s1, Table S1: Univariate analysis of all candidate predictors for bailout stenting; Figure S1: Calibration by deciles: observed versus predicted probability of bailout stenting.

## Abbreviations

The following abbreviations are used in this manuscript:

ACS	Acute coronary syndrome
AUC	Area under the receiver operating characteristics curve
CABG	coronary artery bypass grafting
CAD	coronary artery disease
CCS	chronic coronary syndrome
CI	confidence interval
CKD	chronic kidney disease
DCB	drug-coated balloon
DES	drug-eluting stent
FFR	Fractional flow reserve
ISR	In-stent restenosis
LAD	left anterior descending artery
LCx	left circumflex artery
LVEF	left ventricular ejection fraction
MACE	major adverse cardiovascular events
MI	myocardial infarction
OR	odds ratio
PCI	percutaneous coronary intervention
QCA	quantitative coronary angiography
RCA	right coronary artery
RVD	reference vessel diameter
SD	standard deviation
SYNTAX	Synergy between PCI with TAXUS and cardiac surgery.
vFFR	Vessel fractional flow reserve
