# Supplementary material for: Development and Internal Validation of a Bailout Risk Score in PCI with Drug-Coated Balloons
Source: J Clin Med. 2026 Jan 19;15(2):813. doi: 10.3390/jcm15020813 (PMC12841662; doi:10.3390/jcm15020813)
Supplement: Supplementary file 1 [file jcm-15-00813-s001.zip › jcm-4074860-supplementary.pdf]

Supplementary Materials

Development and internal validation of a Bail-out risk score in PCI with drug-coated balloons.

Table of Contents

- Table S1
- Figure S1

Table S1. Univariate analysis of all candidate predictors for bail-out stenting.

Variable	Reference	Odds Ratio (95% CI)	p-value
**Age (years)**	— (per 1-year ↑)	0.997 (0.967–1.03)	0.844
**Sex**	Female	1.06 (0.543–2.07)	0.863
**BMI (kg/m²)**	— (per 1-unit ↑)	1.0 (0.942–1.07)	0.886
**Hypertension**	Yes	0.892 (0.412–1.93)	0.772
**Lipid-lowering therapy**	Yes	0.481 (0.234–0.987)	**0.046**
**Diabetes**	Yes	1.41 (0.805–2.46)	0.231
**Current smoking**	Yes	0.702 (0.340–1.45)	0.341
**Prior CABG**	Yes	3.30 (1.40–7.75)	**0.006**
**Prior PCI**	Yes	0.407 (0.225–0.739)	**0.003**
**Prior MI**	Yes	0.091 (0.012–0.673)	**0.019**
**eGFR (mL/min/1.73m²)**	— (per 1-unit ↑)	1.0 (0.991–1.01)	0.818
**CKD**	Yes	0.590 (0.278–1.25)	0.169
**LVEF (%)**	— (per 1-unit ↑)	0.968 (0.920–1.02)	0.211
**Clinical presentation**	ACS	3.01 (0.538–16.8)	0.210
**LAD**	Yes	1.64 (0.939–2.88)	**0.082**
**LCx**	Yes	0.463 (0.160–1.34)	0.155

**RCA**	Yes	0.905 (0.486–1.68)	0.753
**Side Branch**	Yes	1.06 (0.386-2.91)	0.910
**Segment**	Proximal	2.48 (1.27–4.86)	**0.008**
**Focal vs Diffuse Disease**	Diffuse	1.96 (1.07–3.59)	**0.029**
**Maximal lesion size (mm)**	— (per 1-mm ↑)	0.642 (0.352–1.17)	0.148
**Bifurcation**	Yes	1.61 (0.727–3.55)	0.241
**Calcification**	Moderate–Severe	1.44 (0.820–2.53)	0.204
**Debulking**	Yes	0.634 (0.186–2.16)	0.467
**Predilation / DCB diameter**	Yes	1.66 (0.096–28.3)	0.728
**DCB inflation pressure (atm)**	— (per 1-atm ↑)	0.928 (0.830–1.04)	0.192
**CTO**	Yes	0.637 (0.241–1.68)	0.365
**Predilation pressure (atm)**	— (per 1-atm ↑)	1.000 (0.926–1.09)	0.930
**Intravascular imaging**	Yes	3.67 (0.852–15.77)	**0.081**

ACS indicates acute coronary syndrome; BMI, body mass index; CABG, coronary artery bypass grafting; CKD, chronic kidney disease; CTO, chronic total occlusion; DCB, drug-coated balloon; eGFR, estimated glomerular filtration rate; LAD, left anterior descending coronary artery; LCX, left circumflex coronary artery; LVEF, left ventricular ejection fraction; MI, myocardial infarction; PCI, percutaneous coronary intervention; and RCA, right coronary artery.

Figure S1. Calibration by deciles: observed versus predicted probability of bail-out stenting.

Decile	N	Observed	Predicted	Observed Percent	Predicted Percent
1	40	0.025	0.0315	2.5	3.1
2	40	0.05	0.0559	5	5.6
3	40	0.075	0.0656	7.5	6.6
4	40	0.075	0.0785	7.5	7.9
5	40	0.075	0.0997	7.5	10
6	40	0.15	0.127	15	12.7
7	40	0.175	0.155	17.5	15.5
8	40	0.15	0.199	15	19.9
9	40	0.25	0.237	25	23.7
10	39	0.436	0.411	43.6	41.1

The study population was divided into ten groups according to increasing predicted probability of bailout stenting. For each decile, the mean predicted probability was compared with the corresponding observed event rate. Overall, predicted and observed probabilities showed good agreement across deciles, with a progressive increase in event rates and no major systematic over- or underestimation, supporting adequate model calibration.
